# Investigating the Effects of Atrial Natriuretic Peptide on the Maternal Endothelium to Determine Potential Implications for Preeclampsia

**DOI:** 10.3390/ijms24076182

**Published:** 2023-03-24

**Authors:** Natalie K. Binder, Sally Beard, Natasha de Alwis, Bianca R. Fato, Tuong-Vi Nguyen, Tu’uhevaha J. Kaitu’u-Lino, Natalie J. Hannan

**Affiliations:** 1 Therapeutics Discovery and Vascular Function in Pregnancy Laboratory, Heidelberg, VIC 3084, Australia; 2 Department of Obstetrics and Gynaecology, University of Melbourne, Heidelberg, VIC 3084, Australia; 3 Mercy Perinatal, Mercy Hospital for Women, Heidelberg, VIC 3084, Australia; 4 Diagnostics Discovery and Reverse Translation Laboratory, Heidelberg, VIC 3084, Australia

**Keywords:** preeclampsia, corin, atrial natriuretic peptide, ANP, cardiovascular disease, endothelium, omental arteries

## Abstract

Preeclampsia is associated with an increased lifelong risk of cardiovascular disease (CVD). It is not clear whether this is induced by persistent systemic organ and vascular damage following preeclampsia or due to a predisposition to both conditions that share cardiovascular pathophysiology. Common to both CVD and preeclampsia is the dysregulation of corin and its proteolytic product, atrial natriuretic peptide (ANP). ANP, a hypotensive hormone converted from pro-ANP by corin, is involved in blood pressure homeostasis. While corin is predominantly a cardiac enzyme, both corin and pro-ANP are significantly upregulated in the gravid uterus and dysregulated in preeclampsia. Relatively little is known about ANP function in the endothelium during a pregnancy complicated by preeclampsia. Here, we investigated the effect of ANP on endothelial cell proliferation and migration, markers of endothelial dysfunction, and receptor expression in omental arteries exposed to circulating preeclamptic toxins. ANP receptor expression is significantly upregulated in preeclamptic vasculature but not because of exposure to preeclampsia toxins tumour necrosis factor α or soluble fms-like tyrosine kinase-1. The supplementation of endothelial cells with ANP did not promote proliferation or migration, nor did ANP improve markers of endothelial dysfunction. The role of ANP in preeclampsia is unlikely to be via endothelial pathways.

## 1. Introduction

Preeclampsia is a disease of pregnancy characterised by new-onset hypertension and multiorgan injury after 20 weeks gestation. Affecting 3–8% of pregnancies around the world [1,2], preeclampsia is a major cause of both fetal and maternal morbidity and mortality [3,4]. While the aetiology of preeclampsia has not been conclusively defined, placental dysfunction leading to placental ischemic injury and hypoxia is believed to have a key role in disease initiation. The hypoxic placenta releases excess levels of anti-angiogenic and pro-inflammatory factors, resulting in widespread maternal endothelial dysfunction and systemic vasoconstriction [5,6]. Women who have experienced a pregnancy complicated by preeclampsia are at an increased long-term risk of developing cardiovascular disease (CVD) [7,8,9,10,11,12]. It is not clear whether the association between preeclampsia and CVD can be ascribed to systemic injury or toxin exposure during the preeclamptic pregnancy or whether women inherently at high risk for CVD are also at high risk for preeclampsia due to shared pathophysiology [13,14].

Common to both preeclampsia and CVD is the dysregulation of corin [15,16,17,18,19,20]. Corin is a cardiac transmembrane serine protease that converts pro-atrial natriuretic peptide (ANP) to the hypotensive hormone ANP [21,22,23]. ANP is essential for blood pressure and electrolyte homeostasis [24], and circulating levels are a prognostic marker of heart disease and hypertension [25]. Changes in circulating corin levels have been linked to CVD, and corin has potential as a biomarker in predicting the severity and outcome of certain cardiac events [19]. Similarly, mutations in the *corin* gene affecting its proteolytic activity have been identified in women with preeclampsia and are prevalent amongst ethnicities with a high incidence of both preeclampsia and CVD [26]. The evaluation of samples collected from cases of preeclampsia has shown increased corin and pro-ANP in the maternal circulation [27,28,29,30], reduced corin and increased pro-ANP in the uterus [27], and inconsistencies regarding corin levels in the placenta; one study showed increased placental expression and the secretion of corin protein [15], while another found no significant differences at the messenger (m)RNA level [31].

A healthy pregnancy typically coincides with a significant increase in corin and pro-ANP in the gravid uterus, which is dysregulated in cases of preeclampsia [27]. This is particularly poignant given that corin-mediated ANP production promotes trophoblast invasion and spiral artery remodelling in mice and in human cell line experiments [27,32]. Spiral artery remodelling is a crucial step in early placentation and is likely aberrant in preeclampsia [33]. Additionally, pregnant mice deficient in corin or ANP have delayed trophoblast invasion, increased blood pressure, and proteinuria, characteristic of preeclampsia [27], as well as cardiac phenotypes [34]. Rats with pregnancy-induced hypertension have reduced placental corin, coinciding with fetal growth restriction [35]. This preeclampsia-like phenotype persists in uterine-specific corin knockout mice that retain cardiac corin expression.

While there is mounting evidence regarding the importance of corin and ANP for spiral artery remodelling in early placentation, corin and ANP are also important regulators of blood pressure homeostasis; ANP regulates blood volume and vascular tone via its actions on the endothelium as a vasodilator [36]. However, far less is known about the role of corin and ANP in endothelial function during a preeclamptic pregnancy [37]. Here, we investigated whether its action occurs through the endothelium, assessing the effect of ANP on endothelial cell proliferation and migration, markers of endothelial dysfunction, and receptor expression in omental arteries exposed to aberrantly produced circulating factors. We have focused on preterm preeclampsia, as this early-onset form of the disease has the greatest association with cardiovascular disease and is thus more likely to involve corin/ANP [38].

## 2. Results

### 2.1. Circulating Levels of Corin and NT-proANP Increased with Preeclampsia

**Figure 1:**
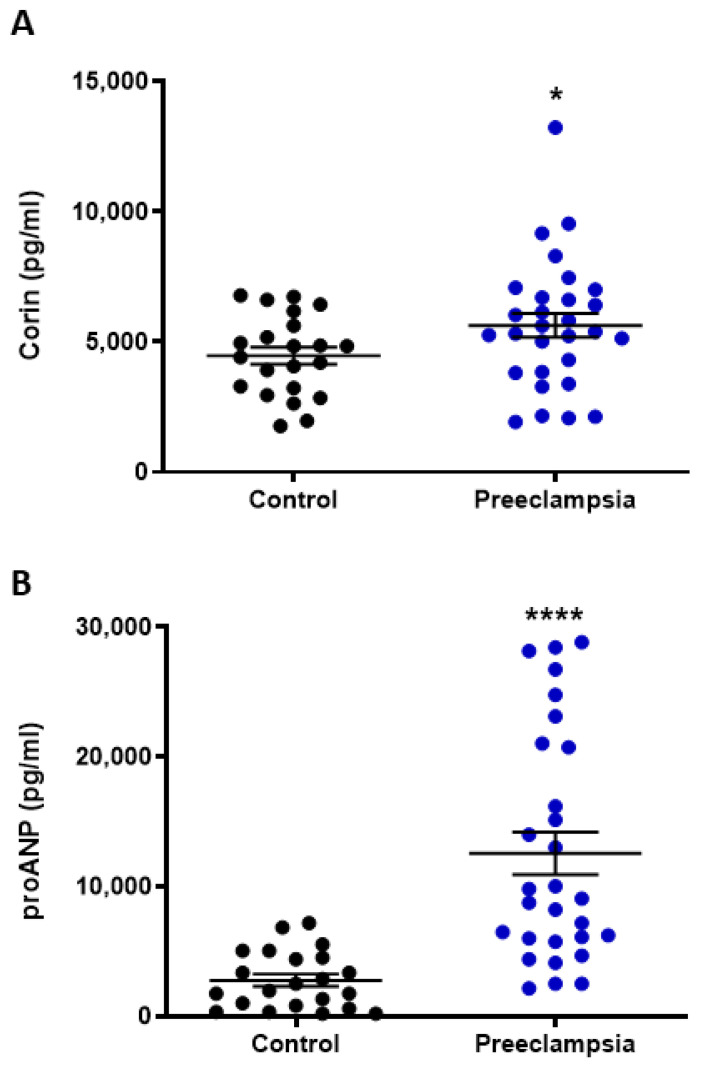
Circulating levels of corin and NT-proANP increased with preeclampsia. In pregnancies complicated by preterm preeclampsia (delivery <34 weeks gestation), there was a significant increase in plasma concentrations of corin (**A**) and N-terminal (NT)-proANP (**B**) compared to gestation-matched (normotensive) controls. Data are mean ± SEM, n = 22–29, * *p* < 0.05, **** *p* < 0.0001.

Compared to gestation-matched normotensive controls, the circulating plasma levels of both corin (*p* < 0.05, Figure 1A) and N-terminal (NT)-proANP (*p* < 0.0001, Figure 1B) were significantly elevated in pregnancies complicated by preterm preeclampsia requiring delivery before 34 weeks of gestation.

**Figure 2:**
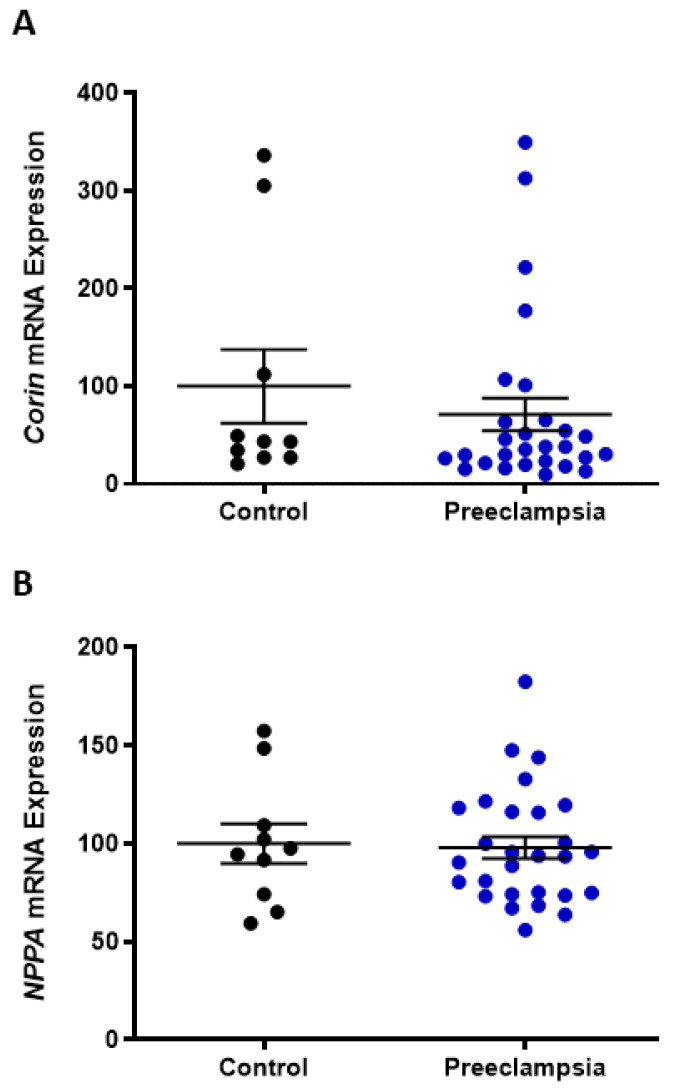
Placental *corin* and *NPPA* (pro-ANP-encoding gene) expression are not altered with preeclampsia. In preterm pregnancies (<34 weeks gestation), placental *corin* (**A**) and *NPPA* (**B**) mRNA expression was not significantly altered between pregnancies complicated by preeclampsia and normotensive controls. Data are mean ± SEM, n = 10–28.

Within the placenta, the mRNA expression of *corin* and the ANP-encoding gene, natriuretic peptide precursor A (*NPPA*), did not significantly differ between pregnancies complicated by preterm preeclampsia or gestation-matched normotensive controls (Figure 2A,B).

### 2.2. Omental Artery Expression of ANP Receptor, Natriuretic Peptide Receptor A (NPR1), Significantly Increased with Preeclampsia

**Figure 3:**
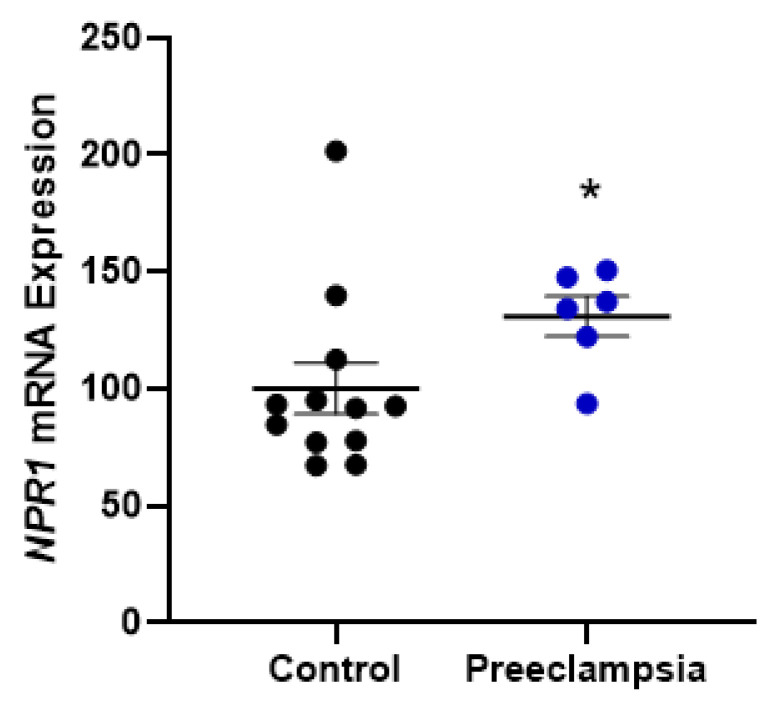
Omental artery expression of ANP receptor *NPR1* significantly increased with preeclampsia. Omental artery expression of *NPR1* is significantly increased in cases of preterm preeclampsia (<34 weeks gestation) compared to normotensive controls. Data are mean ± SEM, n = 6–12, * *p* < 0.05.

The mRNA expression of *NPR1* was significantly higher in omental arteries from pregnancies complicated by preterm preeclampsia compared to normotensive controls delivering an appropriately grown baby at term (*p* > 0.05 Figure 3).

**Figure 4:**
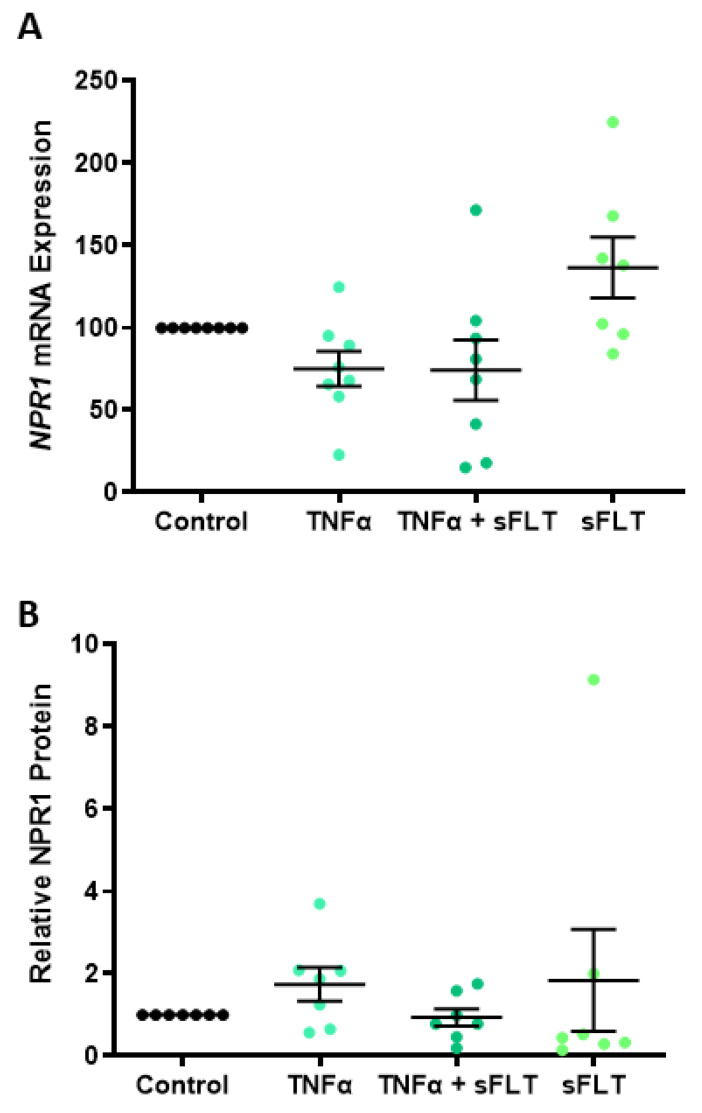
*NPR1* expression and protein in cultured omental arteries are not affected by TNFα or sFlt-1. In cultured omental arteries, *NRP1* mRNA expression (**A**) and protein levels (**B**) were unaltered by treatment with 10 ng/mL TNFα or 250 ng/mL recombinant human sFlt-1 alone or in combination. Data are mean ± SEM, n = 7–8.

Given the observation that soluble fms-like tyrosine kinase (sFlt)-1 and tumour necrosis factor (TNF)α are implicated in the endothelial dysfunction associated with preeclampsia [39,40,41], we investigated their effect on cultured normotensive term omental arteries. Neither *NRP1* mRNA expression (Figure 4A) nor protein levels (Figure 4B) were regulated by TNFα or sFlt-1, alone or in combination.

### 2.3. ANP Increases Heme Oxygenase (HO)-1 Expression but Does Not Affect Human Umbilical Vein Endothelial Cell (HUVEC) Proliferation or Migration

**Figure 5:**
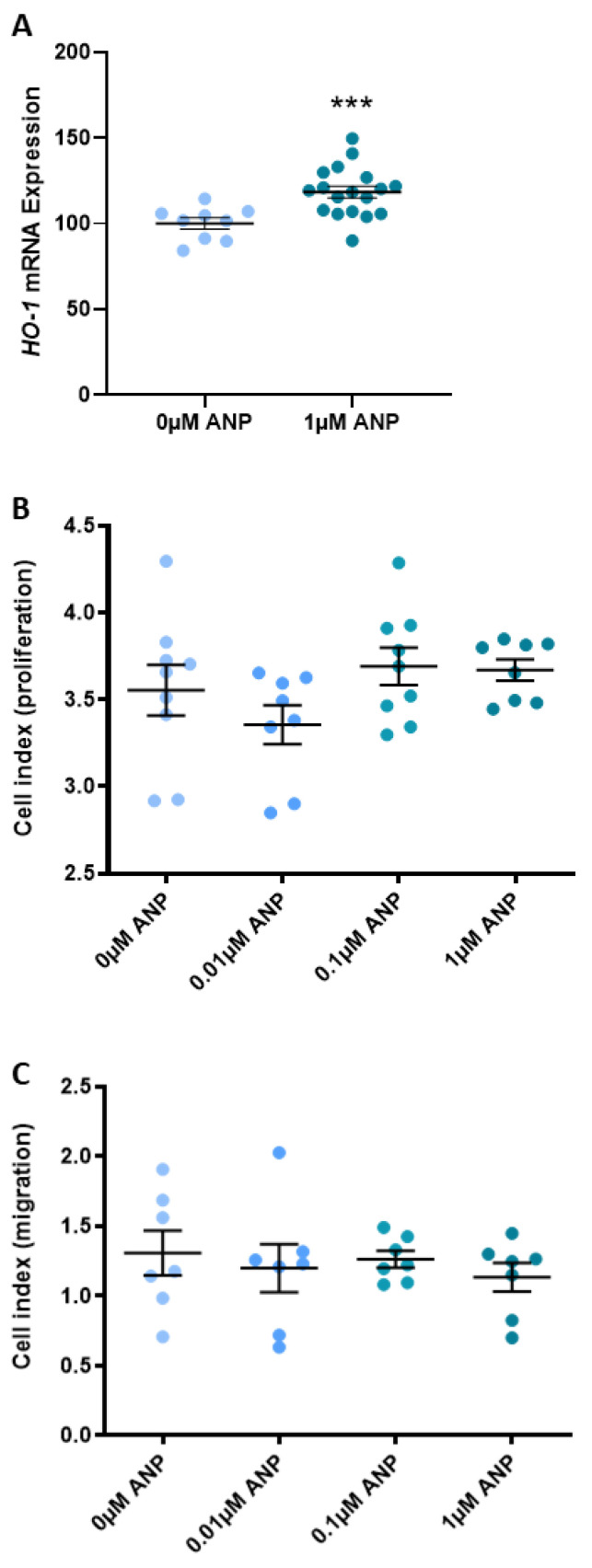
ANP increases *HO-1* expression but does not affect HUVEC proliferation or migration. In isolated primary HUVEC, 1 µM ANP significantly increases *HO-1* mRNA expression compared to 0 µM ANP control (**A**). Incrementally increasing doses of ANP did not affect HUVEC proliferation (**B**) or migration (**C**) compared to 0 µM ANP control. Data are mean ± SEM, n = 7–9, *** *p* < 0.001.

Known for its critical role in the prevention of vascular inflammation, *HO-1* mRNA expression in isolated primary HUVEC is significantly increased with ANP treatments compared to the vehicle control (0 µM ANP, *p* < 0.001; Figure 5A). However, incrementally increasing doses of ANP did not affect HUVEC proliferation (Figure 5B) or migration (Figure 5C) compared to the vehicle control.

**Figure 6:**
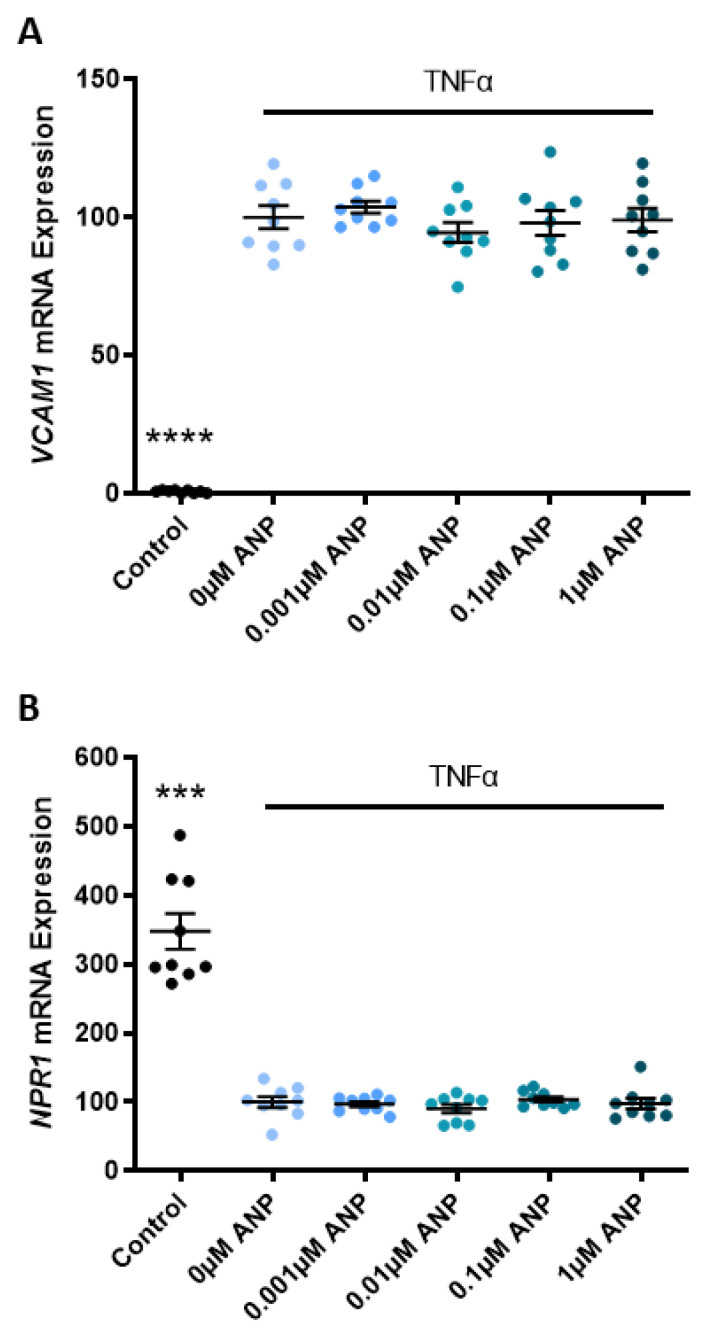
ANP does not reduce the expression of endothelial dysfunction marker *VCAM1* in a model of endothelial dysfunction. In a model of endothelial dysfunction, TNFα significantly increases the expression of *VCAM1* mRNA (**A**) and significantly decreases the expression of *NPR1* mRNA (**B**) in HUVECs. Incrementally increasing doses of ANP did not mitigate the TNFα-induced change in *VCAM1* (**A**) or *NPR1* (**B**) mRNA expression. Data are mean ± SEM, n = 9, *** *p* < 0.001, **** *p* < 0.0001.

In a model of endothelial dysfunction, TNFα significantly increased the expression of vascular cell adhesion molecule (*VCAM)1* mRNA (*p* < 0.0001; Figure 6A) and significantly decreased the expression of *NPR1* mRNA (*p* < 0.001; Figure 6B) in primary HUVEC. Incrementally increasing doses of ANP did not mitigate the TNFα-induced change in *VCAM1* or *NPR1* mRNA expression (Figure 6A,B, respectively).

## 3. Discussion

We have previously identified corin expression in endometrium receptive to embryo implantation and first-trimester implantation sites, specifically localised to the maternal decidual cells surrounding the spiral arteries [31]. These findings support reports from murine studies suggesting a critical role for corin and its proteolytic product, ANP, in trophoblast migration and spiral artery remodelling [27,42]. Defective spiral artery remodelling resulting in placental ischemic injury and hypoxia is believed to be key to the pathogenesis of preeclampsia. Relatively little, however, is known about the role of ANP in endothelial function during preeclampsia.

A major focus of this study was to investigate if the hypotensive hormone ANP has a direct effect on the endothelium in response to preeclampsia-induced endothelial dysfunction, particularly with respect to endothelial cell proliferation and migration, markers of endothelial dysfunction, and receptor expression in omental arteries exposed to circulating preeclamptic toxins. Here, we demonstrate that the treatment of HUVECs with ANP did not promote proliferation or migration, nor did ANP treatment reduce markers of endothelial dysfunction in in vitro models of dysfunction. ANP receptor expression is significantly upregulated in preeclamptic vasculature, but not because of exposure to preeclampsia toxins TNFα or sFlt-1.

### 3.1. Circulating Corin and Pro-ANP Are Elevated in Preeclamptic Pregnancies

Several reports have identified elevated levels of corin and/or pro-ANP in the serum or plasma of women with preeclampsia at approximately 36 weeks gestation [27,30,43] or earlier as a predictive biomarker in high-risk women [44]. Here, we identified significantly elevated plasma corin and pro-ANP in a carefully collected cohort of cases of preterm preeclampsia (<34 weeks gestation) compared to gestation-matched controls. A study by Degrelle and colleagues has suggested that this increased circulating corin in women with preeclampsia is of placental origin [15]. However, this study was significantly underpowered and failed to distinguish between preterm and term preeclampsia for this analysis. Here, we investigated the expression of *corin* and the gene encoding pro-ANP, *NPPA*, in human placenta collected from cases of preterm preeclampsia (requiring delivery before 34 weeks completed gestation) and normotensive gestation-matched controls. Neither *corin* nor *NPPA* expression was overtly altered in placentas complicated with preeclampsia, which is consistent with our previous report [31].

The increased circulating pro-ANP with preeclampsia is likely a response to increasing blood pressure [45]. However, a corresponding decrease in blood pressure is not observed, suggesting there may be an impairment in the proteolytic action of corin converting pro-ANP to the hypotensive hormone, ANP. There have been identifications of several *corin* mutations amongst women with preeclampsia that may result in decreased enzymatic activity [27,46]. It is possible that decreased corin activity with preeclampsia causes a bottleneck in the pathway of pro-ANP conversion to ANP, resulting in a build-up of pro-ANP due to a lack of conversion and, hence, the absence of a blood-pressure-lowering effect with respect to ANP. Several reports have found circulating levels of ANP to be correspondingly elevated with preeclampsia [47,48], although there is the suggestion that ANP may have altered functions under the preeclamptic pathophysiological environment [49,50].

### 3.2. Omental Artery Expression of the ANP Receptor, NPR1, Is Elevated in Preeclamptic Pregnancies

Similarly, changes in the expression of ANP receptors with preeclampsia could also contribute to changes in the way the corin/ANP system works to regulate blood pressure. Here, the mRNA expression of *NPR1*, one of three natriuretic receptors and the principal receptor of ANP, was significantly upregulated in omental arteries from women with preterm preeclampsia compared to normotensive controls. As omental arteries contribute to systemic vascular resistance, a change in receptor expression could indicate a mechanism for blood pressure dysregulation. Unfortunately, these samples were not matched for gestation. This is a limitation of this finding and should be interpreted with caution, as this may, in part, be a gestation-related expression pattern. It is not clear from the literature whether pro-ANP/ANP, potentially including its receptors, fluctuates across gestation [51,52,53]; hence, further investigation is needed. However, there have been other reports of the dysregulation of vascular ANP receptors with preeclampsia, but again, these samples were not gestationally matched [37]. The importance of these endothelial receptor changes is yet to be elucidated.

Given the significant damage caused to the endothelium by anti-angiogenic and pro-inflammatory mediators (in a preeclamptic pregnancy), we wanted to test if two key factors, TNFα and sFlt-1, regulate the expression of *NPR1* in omental arteries. In culture, neither TNFα or sFlt-1, alone or in combination, affected *NPR1* mRNA expression or protein levels. Interestingly, when we induced dysfunction in HUVECs with TNFα, *NPR1* expression significantly decreased. Treating with increasing concentrations of ANP did not restore *NPR1* expression in the presence of TNFα. Of note, *NPR1* knockout mice experience dysregulated cardiac *ANP* expression [54], suggesting a direct relationship between ligand and receptor expression [55]. The immunoreactivity of *NPR1* should also be considered in the context of preeclampsia and warrants further investigation.

### 3.3. ANP Enhances Endothelial Production of HO-1 but Does Not Affect Proliferation, Migration, or Endothelial Dysfunction

Given the importance of angiogenic balance in preeclampsia and the critical role of corin and ANP in trophoblast migration and spiral artery remodelling, we wanted to assess whether ANP promotes endothelial cell migration and proliferation. Despite ANP significantly increasing endothelial *HO-1* expression, which some studies have demonstrated plays a role in regulating both proliferation and angiogenesis [56,57,58], we show that ANP did not promote HUVEC proliferation or migration. When we induced dysfunction in these endothelial cells with TNFα, vascular cell adhesion molecule 1 (*VCAM1*) expression significantly increased. This was not mitigated by treatment with ANP. Endothelial dysfunction is synonymous with both preeclampsia and CVD, and we hypothesised that this may have been a unifying factor highlighting the importance of corin and ANP between the two pathologies. However, our data do not support this.

## 4. Materials and Methods

### 4.1. Tissue Collection

Ethical approval for this study was obtained from the Mercy Health Human Research Ethics Committee (R11/34 and R14/11). All methods were performed in accordance with the National Health and Medical Research Council’s ethical guidelines. Women presenting to the Mercy Hospital for Women, Heidelberg, Australia, gave informed written consent for tissue collection. Maternal venous blood was obtained prior to 34 weeks completed gestation from pregnancies complicated by preterm preeclampsia (requiring delivery <34 weeks gestation) and gestation-matched healthy controls who later went on to deliver an appropriately grown baby at term (patient characteristics Table 1). Placentas were obtained exclusively at caesarean section from pregnancies complicated by preterm preeclampsia (<34 weeks gestation) and gestation-matched preterm normotensive pregnancies with an appropriately grown for gestational age baby (<34 weeks gestation; patient characteristics in Table 2). Omental fatty tissue was obtained at caesarean section from pregnancies complicated by preterm preeclampsia (<34 weeks gestation) and normotensive term pregnancies that delivered an appropriately grown baby (≥37 weeks gestation up to 41 weeks gestation). Preeclampsia was diagnosed in accordance with the guidelines set by the American College of Obstetrics and Gynecology [59]. Umbilical cords were collected from normal-term pregnancies (≥37 weeks gestation up to 41 weeks gestation) at caesarean section. Tissue samples were collected within 30 min of delivery.

### 4.2. Blood and Placenta Processing

Maternal venous blood was collected into plasma EDTA tubes (BD Biosciences, Franklin Lakes, NJ, USA), inverted gently, and then centrifuged at 1000× *g* for 5 min. Plasma fractions were collected and frozen at −80 °C until assessment by an enzyme-linked immunosorbent assay (ELISA).

Four sites were sampled from individual placentas according to CoLab recommendations [60]. Following the removal of the basal plate and chorionic plate surfaces, samples were washed in sterile phosphate-buffered saline (PBS) and stabilised in RNAlater (Thermo Fisher Scientific, Waltham, MA, USA) before being snap-frozen with liquid nitrogen and stored at −80 °C until processing for RNA extraction.

### 4.3. Isolation and Treatment of Omental Arteries

Omental arteries were selected as they are accessible vasculature from pregnant women, and in the case of preeclampsia, they have been exposed to the circulating toxins responsible for endothelial dysfunction and end-organ injury. Omental arteries were dissected from omental fat biopsies and placed in RNAlater for a minimum of 48 h before being snap-frozen with liquid nitrogen and stored at −80 °C until processing for RNA extraction.

A subset of omental arteries obtained from term normotensive pregnancies was cut into 3 mm lengths and cultured overnight in DMEM high Glutamax (Life Technologies, Carlsbad, CA, USA) containing 10% fetal calf serum and 1% antibiotic-antimycotic (Life Technologies) at 37 °C in 20% O_2_ and 5% CO_2_. Artery segments were treated with 10 ng/mL TNFα (Sigma-Aldrich, St Louis, MO, USA), 250 ng/mL recombinant human sFlt-1 (Sigma-Aldrich), or TNFα and sFlt-1 in combination. Following the overnight culture, artery segments were snap-frozen with liquid nitrogen and stored at −80 °C until processing for protein and RNA extraction.

### 4.4. Isolation and Treatment of Primary HUVECs

The umbilical cord vein was infused with 10 mL (1 mg/mL) of collagenase (Worthington, Lakewood, NJ, USA), and cells were isolated as previously described [61]. HUVECs were cultured in M199 media (Life Technologies) containing 20% fetal calf serum, 1% antibiotic-antimycotic, 1% heparin, and 1% endothelial cell growth factor (Sigma-Aldrich) at 37 °C in 20% O_2_ and 5% CO_2_ and used between passage 2 and 4.

Endothelial dysfunction experiments were undertaken using primary HUVECs isolated from six normotensive term pregnancies. HUVECs were pretreated with 10 ng/mL TNF-α for 2 h before ANP was added at the following concentrations of 0 µM (vehicle control), 0.001, 0.01, 0.1, or 1 µM in the presence of TNF-α for a further 24 h. This concentration range has been shown to induce antioxidant defences (*HO-1* expression) in HUVEC [62] and does not affect cell viability. At the cessation of the experiment, cells were permeabilised with lysis buffer and stored at −80 °C until processing for RNA extraction.

### 4.5. ELISA Analysis

Concentrations of corin and proANP were measured in frozen plasma samples using the DuoSet Human Corin and Human NT-ProANP kits, respectively (R&D systems by Bioscience, Waterloo, Australia), as per the manufacturer’s instructions.

### 4.6. Quantitative RT-PCR Analysis

RNA was extracted from primary HUVECs, placenta, and omental arteries using the RNeasy mini kit (Qiagen, Valencia, CA, USA) and quantified using the Nanodrop ND 1000 spectrophotometer (NanoDrop Technologies Inc., Wilmington, DE, USA). We converted 0.2 μg of RNA to cDNA using the Applied Biosystems High-Capacity cDNA Reverse Transcriptase Kit (Life Technologies) as per the manufacturer’s guidelines.

The gene expression of *VCAM1* (Hs01003372_m1), *HO-1* (Hs01110250_m1), *corin* (Hs00198141_m1), *NPPA* (Hs00383230_g1), *NPR1* (Hs00181445_m1), HUVEC housekeeper *YWHAZ* (Hs01122454_m1), placenta housekeepers *TOP1* (Hs00243257_m1), and *CYC1* (Hs00357717_m1) and omental artery housekeepers *B2M* (Hs00187842_m1) and *Actin* (Hs99999903_m1) (Life Technologies) were quantified by real-time PCR (RT-PCR) on the CFX 384 (Bio-Rad, Hercules, CA, USA) using FAM-labeled Taqman universal PCR mastermix and its specific primer/probe set (Life Technologies) with the following run conditions: 50 °C for 2 min; 95 °C for 10 min; 95 °C for 15 s; and 60 °C for 1 min (40 cycles).

### 4.7. Western Blot Analysis

Following overnight culture, protein was extracted from omental arteries with RIPA lysis buffer and quantitated with a BCA assay. Omental artery protein lysates (13 µg) were separated on 10% polyacrylamide gels with wet transfer to PVDF membranes (Millipore, Billerica, MA, USA). Membranes were blocked in 5% skim milk prior to overnight incubation with the primary antibody NPR1 1:5000 (Anti-NPR-A, ab14356, Abcam, Cambridge, UK) in Can Get Signal (Toyobo, Osaka, Japan). Following incubation in secondary anti-rabbit antibody 1:2500 (Anti-Rabbit IgG, W401, Promega, Madison, WI, USA) in Can Get Signal, the bands were visualised using a chemiluminescence detection system (GE Healthcare Life Sciences, Piscataway, NJ, USA) and ChemiDoc XRS (BioRad). Relative densitometry was determined using QuantityOne software (BioRad). Β-actin loading controls were used for densitometric analysis.

### 4.8. xCELLigence

The xCELLigence Real-Time Cell Analyzer Dual Purpose instrument (Roche Diagnostic, Basel, Switzerland) was used to measure cellular proliferation and the migration of HUVECs following treatments with increasing concentrations of recombinant human ANP. For assessments via a proliferation assay, 5000 cells/well were plated in an E-Plate in media containing 0 µM (vehicle control), 0.01, 0.1, or 1 µM ANP in acetic acid. For assessment via a migration assay, 40,000 cells/well were plated in the top chamber of a CIM-Plate, with media in the wells below containing 0 µM (vehicle control), 0.01, 0.1, or 1 µM ANP in acetic acid as a stimulant. Assays were maintained at 37 °C in 20% O_2_ and 5% CO_2_, and electrical impedance was used to determine proliferation after 48 h culture and migration after 24 h culture.

### 4.9. Statistical Analysis

Experimental assays were run in technical duplicates, and the mean of the raw data was used for statistical analysis. qPCR data are represented as a fold change from the control (normalised to 100) and calculated using the 2^-deltadeltaCT method. Data were tested for normal distribution. Student’s *t*-test with Welch’s correction and a 1-way ANOVA (for parametric data) or Kruskal–Wallis test (for non-parametric data) were used as appropriate. Post hoc analysis was carried out using either Tukey’s (parametric) or Dunn’s test (non-parametric). All data are expressed as the mean ± standard error of the mean (SEM). *p* values < 0.05 were considered significant. Statistical analysis was performed using GraphPad Prism 9 software (GraphPad Software, La Jolla, CA, USA).

## 5. Conclusions

These studies using primary human endothelial cells, placenta, plasma, and omental arteries, both normal and pathological tissue, were unable to demonstrate a significant role for ANP in the regulation of endothelial function and/or dysfunction in preeclampsia.

## Figures and Tables

**Table 1:** Patient characteristics for collected plasma samples.

	Control (n = 22)	Preeclampsia (n = 29)
**Maternal Age** Median (IQR)	31 (28–33)	31 (28–35)
**Gestation at Sampling** Median (IQR)	28.5 (27.4–30.3)	29.6 (28.4–31.4)
**Gestation at Delivery** Median (IQR)	39.6 (38.9–40.7)	30.6 (28.3–31.7) ****
**BMI** (kg/m^2^) Median (IQR)	23.5 (21.8–29.0)	32.0 (25.0–37.3) ***
**Parity** no. (%)		
0	10 (45)	22 (76)
**1**	11 (50)	4 (14)
≥2	1 (5)	3 (10)
**Highest SBP prior to delivery** (mmHg) Median (IQR)	130 (125–131)	174 (170–180) ****
**Highest DBP prior to delivery** (mmHg) Median (IQR)	76 (70–81.3)	103 (100–110) ****
**Birth weight** (g) Median (IQR)	3550 (3158–3700)	1305 (918–1497) ****

Body mass index (BMI) data unavailable for n = 4 preeclampsia. *** *p* < 0.001, **** *p* < 0.0001.

**Table 2:** Patient characteristics for collected placental tissue samples.

	Control (n = 10)	Preeclampsia (n = 28)
**Maternal Age** Median (IQR)	29 (24–36)	31 (27–34)
**Gestation at Delivery** Median (IQR)	29.9 (27.4–32)	30.4 (27.6–31.8)
**BMI** (kg/m^2^) Median (IQR)	28.4 (24.0–40.9)	27.0 (24.5–37.0)
**Parity** no. (%)		
0	2 (20)	18 (64)
1	6 (60)	7 (25)
≥2	2 (20)	3 (11)
**Highest SBP prior to delivery** (mmHg) Median (IQR)	125 (110–130)	175 (160–180) ****
**Highest DBP prior to delivery** (mmHg) Median (IQR)	70 (64–76)	100 (90–110) ****
**Birth weight** (g) Median (IQR)	1454 (941–2011)	1314 (811–1424)

BMI data unavailable for n = 3 control; n = 7 preeclampsia. **** *p* < 0.0001.

## Data Availability

Reasonable requests for access to data generated in this study should be directed to the corresponding author, N.J.H.
